# Use of 15-Valent Pneumococcal Conjugate Vaccine Among U.S. Children: Updated Recommendations of the Advisory Committee on Immunization Practices — United States, 2022

**DOI:** 10.15585/mmwr.mm7137a3

**Published:** 2022-09-16

**Authors:** Miwako Kobayashi, Jennifer L. Farrar, Ryan Gierke, Andrew J. Leidner, Doug Campos-Outcalt, Rebecca L. Morgan, Sarah S. Long, Katherine A. Poehling, Adam L. Cohen, ACIP Pneumococcal Vaccines Work Group, Katherine A. Poehling, Sarah S. Long, Jeffrey Kelman, Lucia Lee, Tina Mongeau, Thomas Weiser, Uzo Chukwuma, Kristina Lu, Mamodikoe Makhene, Lynn Fisher, Mark Sawyer, Jason Goldman, David Nace, Emily Messerli, Elissa Abrams, Aleksandra Wierzbowski, Carol Baker, James McAuley, William Schaffner, Virginia Cane, Doug Campos-Outcalt, Monica M. Farley, Keith Klugman, Rebecca L. Morgan, Arthur Reingold, Lorry Rubin, Cynthia Whitney, Richard K. Zimmerman, CDC Contributors, Emma Accorsi, Alison Albert, Shriya Bhatnagar, Lana Childs, Marc Fischer, Rachel Gorwitz, Angela Jiles, Heidi Moline, Pedro Moro, Chukwuebuka Nsofor, Namrata Prasad, Heather Walker, Jacquline Risalvato, Sarah Schillie

**Affiliations:** ^1^National Center for Immunization and Respiratory Diseases, CDC; ^2^College of Medicine, University of Arizona, Phoenix, Arizona; ^3^Department of Health Research Methods, Evidence and Impact, McMaster University, Hamilton, Ontario, Canada; ^4^College of Medicine, Drexel University, Philadelphia, Pennsylvania; ^5^School of Medicine, Wake Forest University, Winston-Salem, North Carolina.; Wake Forest School of Medicine; Drexel University College of Medicine; Center for Medicare & Medicaid Services; Food and Drug Administration; Indian Health Service; National Institutes of Health; American Academy of Family Physicians; American Academy of Pediatrics, Committee on Infectious Diseases; American College of Physicians; American Geriatrics Society, The Society for Post-Acute and Long-term Care Medicine; Association of Immunization Managers; Canadian National Advisory Committee on Immunization; Infectious Diseases Society of America; National Foundation for Infectious Diseases; National Medical Association; University of Arizona; Atlanta Veterans Affairs Medical Center, Emory University; Bill & Melinda Gates Foundation; McMaster University; University of California, Berkeley; Cohen Children’s Medical Center of New York; Emory University; University of Pittsburgh

The 13-valent pneumococcal conjugate vaccine (PCV13 [Prevnar 13, Wyeth Pharmaceuticals, Inc, a subsidiary of Pfizer, Inc]) and the 23-valent pneumococcal polysaccharide vaccine (PPSV23 [Merck Sharp & Dohme LLC]) have been recommended for U.S. children, and the recommendations vary by age group and risk group (*1*,*2*). In 2021, 15-valent pneumococcal conjugate vaccine (PCV15 [Vaxneuvance, Merck Sharp & Dohme LLC]) was licensed for use in adults aged ≥18 years (*3*). On June 17, 2022, the Food and Drug Administration (FDA) approved an expanded usage for PCV15 to include persons aged 6 weeks–17 years, based on studies that compared antibody responses to PCV15 with those to PCV13 (*4*). PCV15 contains serotypes 22F and 33F (in addition to the PCV13 serotypes) conjugated to CRM197 (genetically detoxified diphtheria toxin). On June 22, 2022, CDC’s Advisory Committee on Immunization Practices (ACIP) recommended use of PCV15 as an option for pneumococcal conjugate vaccination of persons aged <19 years according to currently recommended PCV13 dosing and schedules (*1*,*2*). ACIP employed the Evidence to Recommendation (EtR) Framework,* using the Grading of Recommendations, Assessment, Development and Evaluation (GRADE)^†^ approach to guide its deliberations regarding use of these vaccines. Risk-based recommendations on use of PPSV23 for persons aged 2–18 years with certain underlying medical conditions^§^ that increase the risk for pneumococcal disease have not changed.

The 7-valent pneumococcal conjugate vaccine (PCV7 [Prevnar, Wyeth Pharmaceuticals, Inc.]) was the first pneumococcal conjugate vaccine recommended for U.S. children in 2000 and was replaced by PCV13 in 2010. PCV13 was licensed by FDA based on safety and immunogenicity data compared with PCV7, and systematic reviews have shown that PCV13 is effective against acute otitis media, pneumonia, and invasive pneumococcal disease (IPD) in children (*5*–*7*). PCV13 has been recommended for routine use among all children aged 2–59 months. In addition, risk-based use of PCV13 is recommended for children aged 60–71 months with certain underlying medical conditions that increase the risk for pneumococcal disease (hereafter, risk conditions), and for persons aged 6–18 years with an immunocompromising condition,^¶^ cerebrospinal fluid leak, or cochlear implant (a subset of risk conditions). PPSV23 is only recommended for persons aged 2–18 years with risk conditions.

During February–June 2022, ACIP reviewed the epidemiology of pneumococcal disease and considerations for use of PCV15 in children. The ACIP Pneumococcal Vaccines Work Group evaluated the quality of evidence for PCV15 immunogenicity and safety, using the GRADE approach. Applying the EtR Framework, the Work Group reviewed relevant scientific evidence regarding the benefits and harms of PCV15 use among children who are recommended to receive PCV13. Within the EtR framework, ACIP considered the importance of the public health problem, benefits and harms, the target population’s values and preferences, resource use, equity, acceptability, and feasibility of PCV15 use. After a systematic review of the literature, the Work Group defined critical outcomes and used GRADE to assess certainty of evidence rated on a scale of 1 (high certainty) to 4 (very low certainty).**

## Evidence

### Pneumococcal Disease Incidence in Persons Aged <19 Years

Acute otitis media is one of the most common diagnoses associated with outpatient pediatric medical visits (*8*) and antibiotic prescribing (*9*). According to a recent analysis using administrative data, 20,800 all-cause acute otitis media episodes per 100,000 person-years occurred among U.S. persons aged <18 years during 2018, with a higher incidence in younger age groups (*10*). During 2015–2019, in a cohort of 319 U.S. children aged 6–36 months with clinically diagnosed acute otitis media, *Streptococcus pneumoniae* was detected in the middle ear fluid of 24% (*11*); 9% of these children were infected with a PCV13 serotype (including 6C), and 8% with one of the serotypes included in PCV15 but not in PCV13 (serotypes 22F and 33F) (*11*). Additional analysis using administrative data estimated that among persons aged <18 years, 1,280 to 3,990 episodes of health care utilization per 100,000 person-years occurred in 2014 for all-cause pneumonia (*12*), and that during 2018–2019, 87 to 680 hospitalizations per 100,000 population occurred for all-cause pneumonia (*13*). Using population-based surveillance data, *S. pneumoniae* was detected in 4% of persons aged <18 years who were hospitalized with community-acquired pneumonia; the attributable proportion of pneumococcus and serotype distribution among all-cause pneumonia in children and adolescents, however, has not been determined (*14*). According to U.S. multistate surveillance, the incidence of IPD^††^ during 2018–2019 was 7.2 per 100,000 children aged <5 years and 1.5 per 100,000 persons aged 5–18 years. PCV13-serotypes accounted for 21% and 34% of IPD cases in children aged <5 years and persons aged 5–18 years, respectively; similarly, additional serotypes unique to PCV15^§§^ caused 15% and 23% of IPD in children aged <5 years and persons aged 5–18 years, respectively (*15*).

### PCV15 Immunogenicity

Phase II and III randomized controlled trials (RCTs) evaluated the immunogenicity of PCV15 compared with PCV13 in healthy infants and children (*16*–*19*), persons aged 5–17 years with sickle cell disease (*20*), and persons aged 6–17 years living with HIV infection (*21*). The following outcomes were measured 30 days after administration of ≥1 doses of PCV, as specified in the respective study protocols: serotype-specific immunoglobulin G (IgG) geometric mean concentration (GMC) (*16*–2*1*), proportion of participants meeting the serotype-specific IgG value of ≥0.35 *μ*g/mL (response rate) (*16*–*19*), and opsonophagocytic activity geometric mean titer in a subset of the study population (*17**,**20**,**21*). One of the phase III RCTs enrolled healthy children aged 42–90 days who received PCV13 or PCV15 at ages 2, 4, 6, and 12–15 months. Except for serotype 6A GMC ratio after dose 3, PCV15 met criteria for noninferiority^¶¶^ to PCV13 for the 13 shared serotypes regarding the response rate after dose 3 and GMC ratio after dose 3 and after dose 4. PCV15 elicited statistically significantly higher immune response for serotype 3 than for PCV13 (*17*). PCV15 met the noninferiority criteria compared with PCV13 for the two unique serotypes 22F and 33F (*17*).

Another phase III RCT enrolled healthy children aged 42–90 days who were randomized to five different arms that received 0–4 doses of PCV15 in combination with PCV13 to complete their 4-dose PCV series, to assess interchangeable use of PCV13 and PCV15 (*19*). IgG GMCs for the 13 shared serotypes measured after dose 4 in children who received ≥1 dose of PCV15 were generally comparable to those in children who completed their PCV series with PCV13 only. Among PCV-naïve or partially vaccinated persons aged 7 months–17 years who received catch-up PCV doses, PCV15 elicited IgG GMCs comparable to PCV13 for the 13 shared serotypes (*18*). Among children with sickle cell disease, a dose of PCV15 elicited higher IgG GMC for six of 13 shared serotypes and for the two unique serotypes (*20*). Among children living with HIV infection, a dose of PCV15 elicited higher IgG GMC for eight of 13 shared serotypes and for the two unique serotypes, compared with a dose of PCV13; 1 dose of PCV15 followed by PPSV23 8 weeks later elicited higher IgG GMC for three of 13 shared serotypes compared with a dose of PCV13 followed by PPSV23, although IgG GMC for 22F and 33F were lower in those who received PCV15 followed by PPSV23 than in those who received PCV13 followed by PPSV23 (*21*).

### PCV15 Safety

Safety of PCV15 was assessed in seven RCTs with 4,778 persons aged 6 weeks–17 years who received ≥1 dose of PCV15 (*16*–*23*). Two of these RCTs that enrolled children and adolescents with sickle cell disease or HIV infection were assessed separately. Of the remaining five studies that enrolled healthy children, four were also included in the immunogenicity assessment (*16*–*19*). Three studies included preterm infants born at <37 weeks gestation (*17*,*19*,*23*). Across these five studies, four of 4,540 children who received PCV15 developed serious adverse events*** that were considered to be vaccine-related, compared with one of 2,655 children who received PCV13. The two RCTs that enrolled children with sickle cell disease or HIV infection were both included in the immunogenicity assessment (*20**,**21*). No serious adverse events that were considered to be vaccine-related were reported in either study.

Given the similarities in the target population and the vaccine schedule used, a detailed safety assessment was performed combining data from three studies of healthy infants who received 4 doses of PCV15 (3,002) or PCV13 (1,467) at ages 2, 4, 6, and 12–15 months (*17*,*19*,*22**,**23*). The most commonly reported adverse events after any PCV dose included irritability (75.1% in the PCV15 group versus 72.7% in the PCV13 group), somnolence (56.7% versus 59.3%), injection site pain (45.1% versus.43.5%), and decreased appetite (39.1% versus 36.0%). Febrile convulsions were reported in eight of 3,002 (0.3%) children who received PCV15, and three of 1,467 (0.2%) who received PCV13. Nearly all (8 of 11, 73%) febrile convulsions occurred ≥50 days after PCV receipt, and none were deemed vaccine-related by study investigators. Adverse events that were considered to be vaccine-related were reported in 89.1% of children who received PCV15 and 86.4% of those in the PCV13 group. Two children (0.1%) who received PCV15 and none who received PCV13 had serious adverse events that were considered to be vaccine-related; both of these children were hospitalized for fever after vaccine administration (after dose 1 and after dose 3). A maximum rectal (or rectal equivalent) temperature of ≥104°F (40°C) within the first 7 days after vaccination was reported for 19 of 2,772 (0.7%) children who received a fourth dose of PCV15 and three of 1,287 (0.2%) who received PCV13.

### Cost-Effectiveness

Two economic models (CDC model and Merck model) that assessed cost-effectiveness compared the use of PCV15 and PCV13 according to the currently recommended PCV13 4-dose series for children aged <2 years (*24*). PCV15 and PCV13 were assumed to have the same vaccine effectiveness against disease caused by the 13 serotypes contained in PCV13. For PCV15, the effectiveness against the two additional serotypes was assumed to be comparable to the overall effectiveness against disease caused by the serotypes contained in PCV13. In the CDC model, PCV15 effectiveness against IPD caused by the two additional serotypes was assumed to be 86% and the effectiveness against IPD caused by most of the other serotypes (excluding serotype 3 and 19F) was assumed to be 86%. Effectiveness against serotypes 3 and 19F disease was assumed to be lower than that against the other PCV serotypes (*25*). In the Merck model, PCV15 effectiveness against IPD caused by the two additional serotypes was assumed to be 86% and the effectiveness against the other serotypes was assumed to range from 80% to 100%. In both models, using PCV15 instead of PCV13 for routine vaccination of children was cost-saving^†††^ in all scenarios examined, including scenarios in which the PCV15 cost per dose**^§§§^** ranged from $4 less to $2 more than the PCV13 cost per dose.

## Summary

PCV15 as an option for pneumococcal conjugate vaccination is expected to reduce pneumococcal disease incidence in children because it induces immunity against additional disease-causing serotypes. Findings from RCTs suggested that the immunogenicity and safety of PCV15 are generally comparable to those of PCV13. Cost-effectiveness studies demonstrated that routine use of PCV15 for children aged <2 years was cost-saving, assuming that the cost and effectiveness of PCV15 for the 13 shared serotypes will remain comparable to those of PCV13 and that PCV15 will provide protection against the two additional serotypes. A summary of Work Group deliberations on use of PCV15 as an option for pneumococcal conjugate vaccination is available in the EtR tables.**^¶¶¶^**

## Recommendations for Use of PCV

ACIP recommends use of PCV (either PCV13 or PCV15) for all children aged 2–59 months. In addition, risk-based PCV use is recommended for children aged 60–71 months with risk conditions, and persons aged 6–18 years with an immunocompromising condition, cerebrospinal fluid leak, or cochlear implant. For all recommendations, PCV13 and PCV15 can be used interchangeably. Interruption of the vaccination schedule does not require reinstitution of the entire series or the addition of extra doses.

### Persons Aged <19 Years with No Previous PCV13 or PCV15 Vaccination

**Infants aged 2–6 months.** Four doses of PCV (either PCV13 or PCV15) are recommended. The primary infant series consists of 3 doses of PCV. Infants receiving their first dose at age ≤6 months should receive 3 doses of PCV at intervals of approximately 8 weeks (with a minimum interval of 4 weeks). The fourth (booster) dose is recommended at age 12–15 months and ≥8 weeks after the third dose (Table 1).

**TABLE 1:** Recommended schedule for use of pneumococcal conjugate vaccine* among previously unvaccinated infants, children, and adolescents, by age at first vaccination and health status — United States, 2022

Age at first vaccination/Health status	Primary PCV13/PCV15 series*^,†^	PCV13/PCV15 booster dose*^,§^
**All children**		
2–6 mos	3 doses	1 dose at 12–15 mos
7–11 mos	2 doses	1 dose at 12–15 mos
12–23 mos	2 doses	Not indicated
**Healthy children**		
24–59 mos	1 dose	Not indicated
**Children with certain underlying medical conditions^¶^**		
24–71 mos	2 doses	Not indicated
**Children and adolescents with an immunocompromising condition,^¶^ cerebrospinal fluid leak, or cochlear implant**		
6–18 yrs	1 dose	Not indicated

**Abbreviations:** PCV = pneumococcal conjugate vaccine; PCV13 = 13-valent PCV; PCV15 = 15-valent PCV.

*Either PCV13 or PCV15 can be used to complete the recommended PCV series.

^†^ Minimum interval between doses is 8 weeks except for children vaccinated at age <12 months, for whom the minimum interval between doses is 4 weeks. The minimum age for administration of first dose is 6 weeks.

^§^ Administered ≥8 weeks after the previous PCV13/PCV15 dose.

^¶^ Certain underlying medical conditions include cerebrospinal fluid leak; chronic heart disease; chronic lung disease; cochlear implant; diabetes mellitus; immunocompromising conditions (chronic renal failure or nephrotic syndrome; congenital or acquired asplenia or splenic dysfunction; congenital or acquired immunodeficiencies; diseases and conditions treated with immunosuppressive drugs or radiation therapy, including malignant neoplasms, leukemias, lymphomas, Hodgkin disease, and solid organ transplant; HIV infection; and sickle cell disease and other hemoglobinopathies). These children are also recommended to receive 23-valent pneumococcal polysaccharide vaccine.

Infants should begin the schedule at age 2 months, although the first dose can be administered as early as 6 weeks. For prematurely born infants (i.e., <37 weeks’ gestation) who are medically stable enough to be vaccinated (*26*), PCV should be administered at the recommended age concurrent with other routine vaccinations.

**Infants aged 7–11 months.** When PCV is initiated at age 7–11 months, 3 doses (either PCV13 or PCV15) are recommended. The first 2 doses should be administered with an interval of ≥4 weeks between doses. The third dose should be administered at age 12–15 months, ≥8 weeks after the second PCV dose.

**Children aged 12–23 months.** When PCV is initiated at 12–23 months of age, 2 doses (either PCV13 or PCV15) are recommended, with an interval of ≥8 weeks between doses.

**Children aged 24–71 months.** Unvaccinated healthy children aged 24–59 months should receive a single dose of PCV (either PCV13 or PCV15). Unvaccinated children aged 24–71 months with any risk condition should receive 2 doses of PCV (either PCV13 or PCV15) with an interval of ≥8 weeks between doses. Routine use of PCV is not recommended for healthy children aged ≥5 years who have not yet received a dose of PCV.

**Children and adolescents aged 6–18 years with an immunocompromising condition, cochlear implant, or cerebrospinal fluid leak.** If a dose of PCV13 or PCV15 has not been administered previously, a single dose of PCV13 or PCV15 is recommended, regardless of whether the child has previously received PPSV23, even if PCV7 was received.

### Persons Aged <19 Years Vaccinated Previously with PCV13 or PCV15 

**Infants and children aged <24 months.** Infants and children aged <24 months who have received ≥1 dose of PCV (either PCV13 or PCV15) should complete the vaccination series with either PCV13 or PCV15 (Table 2).

**TABLE 2:** Recommendations for administering pneumococcal conjugate vaccine* to incompletely vaccinated children, by age at visit, health status, and vaccination history — United States, 2022

Age at visit/Health status	No. of previous PCV13/PCV15 doses received	Recommended PCV13/PCV15 regimen^†^	No. of PCV13/ PCV15 doses to complete series by age 24 mos
All children			
2–6 mos	1	3 additional doses: 2 doses, 8 wks apart; last dose at age 12–15 mos	4
	2	2 additional doses: 1 dose, 8 wks after most recent dose; last dose at age 12–15 mos	4
	3	1 additional dose at age 12–15 mos	4
7–11 mos	1 or 2 (at age <7 mos) or 1 (at age ≥7 mos)	2 additional doses: 1 dose, 8 wks after last dose; last dose ≥8 weeks later, at age 12–15 mos	3 or 4
	3 (at age <7 mos) or 2 (at age ≥7 mos)	1 additional dose at age 12–15 mos	3 or 4
12–23 mos	1 (at age <12 mos)	2 additional doses, ≥8 wks apart	3
	1 (at age ≥12 mos)	1 additional dose, ≥8 wks after most recent dose^†^	2
	2 or 3 (at age <12 mos)	1 additional dose, ≥8 wks after most recent dose	3 or 4
**Healthy children**			
24–59 mos	Any incomplete schedule by 24 mos	1 additional dose, ≥8 wks after most recent dose	NA
5–18 yrs	Any incomplete schedule by 24 mos	No additional dose	NA
**Children with certain underlying medical conditions^§^**			
24–71 mos	Any incomplete schedule^¶^ of<3 doses by age 24 mos	2 doses: first dose ≥8 wks after most recent dose; second dose ≥8 wks later	NA
	3 (all at age <12 mos)	1 dose, ≥8 wks after most recent dose	NA

**Abbreviations**: NA = not applicable; PCV = pneumococcal conjugate vaccine; PCV13 = 13-valent PCV; PCV15 = 15-valent PCV.

* Either PCV13 or PCV15 can be used to complete the recommended PCV series.

^†^ Minimum interval between doses is 8 weeks except for children vaccinated at age <1 year, for whom minimum interval between doses is 4 weeks.

^§^ Certain underlying medical conditions include cerebrospinal fluid leak; chronic heart disease; chronic lung disease; cochlear implant; diabetes mellitus; immunocompromising conditions (chronic renal failure or nephrotic syndrome; congenital or acquired asplenia or splenic dysfunction; congenital or acquired immunodeficiencies; diseases and conditions treated with immunosuppressive drugs or radiation therapy, including malignant neoplasms, leukemias, lymphomas, Hodgkin disease, and solid organ transplant; HIV infection; and sickle cell disease and other hemoglobinopathies). These children are also recommended to receive 23-valent pneumococcal polysaccharide vaccine.

^¶^ See column “No. of PCV13/ PCV15 doses to complete series by age 24 mos” to determine an incomplete schedule of <3 doses by 24 months.

**Children aged 24–71 months.** For all healthy children aged 24–59 months with any incomplete PCV schedule as of age 24 months, 1 additional dose of PCV is recommended. For children aged 24–71 months with any risk conditions who have received any incomplete schedule of <3 PCV doses**** before age 24 months, 2 additional PCV doses of PCV are recommended. Children aged 24–71 months with any risk conditions who have received their 3-dose PCV primary series before age 12 months but have not received their fourth booster dose, are recommended to receive a single additional PCV dose. The minimum interval between doses is 8 weeks.

**Complete PCV13 vaccination.** A supplemental dose of PCV15 is not indicated for children who have received 4 doses of PCV13 or who completed another age-appropriate PCV13 schedule.

### Administration of PPSV23 After PCV13 or PCV15 Among Persons Aged 2–18 Years with Risk Conditions 

Children aged ≥2 years with any risk conditions should receive PPSV23 after completing all recommended PCV doses (either PCV13 or PCV15). These children should receive a single dose of PPSV23 at age ≥2 years and ≥8 weeks after the most recent PCV dose (Table 3). Children who have received PPSV23 but have not yet completed their recommended PCV doses should receive PCV ≥8 weeks after the PPSV23 dose. When elective splenectomy, immunocompromising therapy, or cochlear implant placement is being planned, PCV or PPSV23 vaccination should be completed ≥2 weeks before surgery or initiation of therapy, if possible.

**TABLE 3:** Risk-based pneumococcal vaccine recommendations for children and adolescents with underlying medical conditions that increase the risk for pneumococcal disease — United States, 2022

Risk group/Condition	PCV* for children aged <6 yrs	PCV* for persons aged 6–18 yrs	PPSV23 for children aged ≥2 yrs	
	Recommended	Recommended	Recommended	Single revaccination 5 yrs after first dose
**Immunocompetent children**				
Chronic heart disease^†^	Y	N	Y	N
Chronic lung disease^§^	Y	N	Y	N
Diabetes mellitus	Y	N	Y	N
Cerebrospinal fluid leak	Y	Y	Y	N
Cochlear implant	Y	Y	Y	N
**Children with immunocompromising conditions**				
Chronic renal failure or nephrotic syndrome	Y	Y	Y	Y
Congenital or acquired asplenia, or splenic dysfunction	Y	Y	Y	Y
Congenital or acquired immunodeficiency^¶^	Y	Y	Y	Y
Diseases and conditions treated with immunosuppressive drugs or radiation therapy**	Y	Y	Y	Y
HIV infection	Y	Y	Y	Y
Sickle cell disease or other hemoglobinopathies	Y	Y	Y	Y
Solid organ transplant	Y	Y	Y	Y

**Abbreviations**: N = no; PCV = pneumococcal conjugate vaccine; PCV13 = 13-valent PCV; PCV15 = 15-valent PCV; PPSV23 = 23-valent pneumococcal polysaccharide vaccine; Y = yes.

* Either PCV13 or PCV15 can be used.

^†^ Recommendations are of particular importance for children with cyanotic congenital heart disease and cardiac failure.

^§^ Including asthma if treated with high-dose oral corticosteroid therapy.

^¶^ Includes B-(humoral) or T-lymphocyte deficiency; complement deficiencies, particularly C1, C2, C3, and C4 deficiency; and phagocytic disorders (excluding chronic granulomatous disease).

** Including malignant neoplasms, leukemias, lymphomas, and Hodgkin disease.

**Revaccination with PPSV23 among children with immunocompromising conditions.** Children aged ≥2 years who have an immunocompromising condition should receive a second dose of PPSV23 ≥5 years after the first PPSV23 dose.

**Recipients of hematopoietic stem cell transplants.** Recipients of hematopoietic stem cell transplants are recommended to receive 3 sequential PCV doses followed by a dose of PPSV23 beginning 3–6 months after the transplant, as described in the General Best Practice Guidelines for Immunization (*27*). In children with graft-versus-host disease, PPSV23 can be replaced with a fourth dose of PCV.

### Vaccine Administration

PCV13 and PCV15 are both available in a single-dose prefilled syringe as a 0.5-mL dose administered intramuscularly. Either PCV13 or PCV15 can be administered at the same time as other routine childhood vaccinations, including COVID-19 vaccines (*28*), in separate syringes and using different injection sites. Concurrent PCV15 administration with vaccines containing diphtheria; tetanus; acellular pertussis; inactivated poliovirus; *Haemophilus influenzae* type b; hepatitis A; hepatitis B; measles, mumps, and rubella; rotavirus; and varicella were studied (*17*,*19*). Immunogenicity of these antigens was similar when administered concurrently with PCV15 and PCV13 (*17*,*19*). Coadministration of PCV15 with meningococcal vaccines has not been studied. The same precautions used for coadministration of PCV13 and meningococcal vaccines should be applied when PCV15 is used (*29*). Risk for febrile seizures in children who received PCV15 concurrently with an influenza vaccine has not been studied.

## Reporting of Adverse Events

Before administering PCV or PPSV23, health care providers should consult relevant package inserts regarding precautions and contraindications (*30*–*32*). Adverse events occurring after administration of any vaccine should be reported to the Vaccine Adverse Event Reporting System (VAERS). Reports can be submitted to VAERS online, by fax, or by mail. More information about VAERS is available at https://vaers.hhs.gov.

## Future Research and Monitoring Priorities

CDC and ACIP will continue to assess safety of PCV15; monitor the impact of implementation of new recommendations, including the impact on health equity; and assess postimplementation vaccine effectiveness. CDC and ACIP will update pneumococcal vaccination recommendations as appropriate.

**Summary**

**What is already known about this topic?**

Currently, the 13-valent pneumococcal conjugate vaccine (PCV13) and the 23-valent pneumococcal polysaccharide vaccine (PPSV23) are recommended for U.S. children, and the recommendations vary by age group and risk group.

**What is added by this report?**

On June 22, 2022, the Advisory Committee on Immunization Practices recommended use of PCV15 as an option for pneumococcal conjugate vaccination of persons aged <19 years, according to currently recommended PCV13 dosing and schedules. Risk-based recommendations on use of PPSV23 have not changed.

**What are the implications for public health practice?**

Use of PCV15 as an alternative to PCV13 is expected to further reduce pneumococcal disease incidence in children and adolescents.

## Footnotes

*
https://www.cdc.gov/vaccines/acip/recs/grade/downloads/acip-evidence-recs-framework.pdf

† https://www.cdc.gov/vaccines/acip/recs/grade/about-grade.html^§^ Cerebrospinal fluid leak; chronic heart disease; chronic lung disease; cochlear implant; diabetes mellitus; immunocompromising conditions (chronic renal failure or nephrotic syndrome; congenital or acquired asplenia or splenic dysfunction; congenital or acquired immunodeficiencies; diseases and conditions treated with immunosuppressive drugs or radiation therapy, including malignant neoplasms, leukemias, lymphomas, Hodgkin disease, and solid organ transplant; HIV infection; and sickle cell disease and other hemoglobinopathies).

¶ Chronic renal failure or nephrotic syndrome; congenital or acquired asplenia or splenic dysfunction; congenital or acquired immunodeficiencies; diseases and conditions treated with immunosuppressive drugs or radiation therapy, including malignant neoplasms, leukemias, lymphomas, Hodgkin disease, and solid organ transplant; HIV infection; and sickle cell disease and other hemoglobinopathies.

** https://www.cdc.gov/vaccines/acip/recs/grade/pneumo-PCV15-child.html; https://www.cdc.gov/vaccines/acip/recs/grade/pneumo-PCV15-child-risk-based.html

†† The case definition used by CDC’s Active Bacterial Core surveillance is isolation of *S. pneumoniae* from a normally sterile site or pathogen-specific nucleic acid in a specimen obtained from a normally sterile body site using a validated molecular test. https://www.cdc.gov/abcs/methodology/case-def-ascertain.html

§§ Serotypes 22F and 33F, in addition to PCV13 serotypes.

¶¶ Noninferiority for the 13 shared serotypes with PCV13 requires the lower bound of the 2-sided 95% CI for IgG GMC ratio (V114/PCV13) to be >0.5 (1-sided p<0.025) after dose 3 or after dose 4, or the lower bound of the 2-sided 95% CI for the difference in response rates (V114 −PCV13) to be >−10 percentage points (1-sided p<0.025) after dose 3, where the responders are defined as IgG ≥0.35 *μ*g/mL. Noninferiority for the two unique serotypes 22F and 33F requires the lower bound of the 2-sided 95% CI for the difference in response rates (PCV15-PCV13) after dose 3 or after dose 4 to be >−10 percentage points (1-sided p<0.025) compared with lowest observed response rate in PCV13 excluding serotype 3, or the lower bound of the 2-sided 95% CI for IgG GMC ratio (PCV15/PCV13) compared with lowest observed IgG GMC in PCV13 excluding serotype 3 to be >0.5 (1-sided p<0.025).

*** Serious adverse events were defined as any untoward medical occurrence that, at any dose, resulted in death; was life-threatening; required inpatient hospitalization or prolongation of existing hospitalization; resulted in persistent or significant disability or incapacity; was a congenital anomaly or birth defect; or was another important medical event. https://clinicaltrials.gov/ProvidedDocs/71/NCT03692871/Prot_SAP_000.pdf

††† The use of PCV15 had lower overall costs and improved health outcomes relative to the use of PCV13.

§§§ Cost per dose was a weighted average of public and private dose costs from the CDC vaccine price list and from the information provided by Merck.

¶¶¶ https://www.cdc.gov/vaccines/acip/recs/grade/pneumo-PCV15-child-risk-based-etr.html; https://www.cdc.gov/vaccines/acip/recs/grade/pneumo-PCV15-child-etr.html

**** Certain children would be considered to have received a complete schedule, even if the total number of PCV doses received by 24 months is <3 doses; an example is a child who received 2 doses of PCV during age 12–23 months.
